# Genome-Wide Identification of the Glycosyltransferase 4 Family and Characterization of *GmSPS8* Associated with Seed Weight in Soybean

**DOI:** 10.3390/cimb48090918

**Published:** 2026-09-08

**Authors:** Hongxiang Cao, Luqi Liu, Kaibo Zhang, Su Ma, Miao Wang, Yongjing Sun, Yongbin Zhuang, Baoyin Chen, Jinfei Zhang, Dajian Zhang, Xiaoming Li

**Affiliations:** College of Agronomy, Shandong Agricultural University, Tai’an 271018, China; 15006367518@163.com (H.C.); 2022110050@sdau.edu.cn (L.L.); cnsdzkb@163.com (K.Z.); ms17662591280@163.com (S.M.); 15255643783@163.com (M.W.); 18366333083@163.com (Y.S.); zyb1986@sdau.edu.cn (Y.Z.); bychen@sdau.edu.cn (B.C.); jfzhang@sdau.edu.cn (J.Z.); dajianzhang@sdau.edu.cn (D.Z.)

**Keywords:** glycosyltransferase 4, 100-seed weight, seed development, sucrose phosphate synthase, sucrose synthase

## Abstract

Glycosyltransferases (GTs) are key enzymes that catalyze the transfer of sugar moieties to diverse acceptor molecules and play important roles in a wide range of biological processes, including plant growth, development, and environmental adaptation. However, glycosyltransferase family 4 (GT4), the second-largest GT family, remains poorly characterized in soybean. In this study, we identified 60 GT4 family members in the soybean genome based on sequence information from the CAZy database and systematically characterized their phylogenetic relationships, genomic organization, and expression patterns by integrating genomic and multidimensional transcriptomic data. Furthermore, using a previously established soybean ethyl methanesulfonate (EMS) mutant population, we identified *GmSPS8* as a candidate gene potentially involved in the regulation of seed weight. Notably, natural variation and haplotype analyses revealed that GmSPS8 is located within a genomic region subject to domestication selection, suggesting its potential involvement in the evolutionary selection of soybean seed size. In sum, we systematically characterized the soybean GT4 gene family and identified *GmSPS8* as a potential domestication-associated candidate gene for seed weight regulation, providing new insights into the functional characterization of soybean GT4 family members and the identification and utilization of genes associated with soybean yield-related traits.

## 1. Introduction

Glycosyltransferases (GTs; EC 2.4.x.y) are essential metabolic enzymes found in various organisms. They facilitate glycosylation reactions by transferring sugar moieties from donor molecules to a range of acceptors, including sugars, lipids, proteins, and nucleic acids, thereby forming glycosidic bonds [1]. Traditionally, in accordance with the guidelines set forth by the International Union of Biochemistry and Molecular Biology (IUBMB), GTs have been classified based on the specificity of their donors, acceptors, and products [2]. For instance, UDP-glycosyltransferases (UGTs) utilize UDP-glucose as the sugar donor. Furthermore, GTs can also be categorized according to the types of glycosidic bonds they form, including O-type, S-type, N-type, and C-type glycosyltransferases [3]. While these classification methods are simple and intuitive, advancements in genomics have led to the characterization of an increasing number of GT family members, rendering traditional classifications inadequate for the post-genomic era [4]. To address these limitations, Campbell et al. proposed a novel approach to classify GT families based on amino acid sequence similarity [5]. Currently, classification methods that integrate donor and acceptor characteristics, product features, and amino acid sequences are gaining acceptance and are being incorporated into the Carbohydrate-Active enZYmes Database (CAZy) [6].

Glycosyltransferase-mediated glycosylation modifications play crucial roles in various biological processes. First, glycosyltransferases (GTs) serve as essential enzymes in the synthesis of polysaccharides, glycolipids, and glycoproteins. Compared to animals, plants possess a greater abundance of polysaccharides, such as cellulose, and glycosylated products, resulting in a higher number of GTs [1]. For example, cellulose synthase (CESA), a member of the GT2 family, functions as the primary enzyme responsible for synthesizing cell wall cellulose [7]. Second, GTs facilitate the precise regulation of hormone levels by modifying endogenous hormones [8]. In *Arabidopsis thaliana*, UGT74D1 mediates the inactivation of endogenous indole-3-acetic acid (IAA) through the glycosylation of OxIAA, thereby regulating IAA levels [9]. Additionally, members of the UGT74F family assist in the sink of salicylic acid by glycosylating salicylic acid (SA), which enables rapid adjustments to salicylic acid levels under specific stress conditions [10]. GTs are involved in biotic and abiotic stress through various metabolic pathways. UGT73C3 and UGT73C4 enhance the immune capacity of *A*. *thaliana* by catalyzing the glycosylation of resin alcohols [11]. In corn, UGTs Bx8 and Bx9 defend against biological stress by glycosylating phenoxy acid compounds to produce toxic glycosides [12]. Furthermore, UGT83A1 improves drought resistance, salt tolerance, and heat tolerance in rice by regulating the synthesis of lignin and flavonoids [13]. The overexpression of UGT76E11 increases the scavenging ability of reactive oxygen species by elevating the levels of free flavonoids, such as quercetin, naringin, and kaempferol, in *A*. *thaliana* [14]. Additionally, GTs, particularly UGTs, play a crucial role in the detoxification of both exogenous and endogenous compounds [15].

Currently, the CAZy database includes a total of 140 glycosyltransferase (GT) gene families, with GT1 and GT4 representing the two largest families. The GT1 gene family, often referred to as the UGT gene family, primarily utilizes UDP-Glc as the glycogen donor. In botanical research, UGTs have garnered significant attention, particularly for their well-documented roles in the anabolic metabolism of lignin and flavonoids [16,17]. As the second-largest glycosyltransferase (GT) family, the GT4 family comprises multiple members, including SUS, SPS, GBSS, and DGD. SUS and SPS are two major constituents of the GT4 family and play crucial roles in sucrose synthesis and metabolism. As a key regulatory enzyme in plant sucrose synthesis, SPS activity is modulated by mechanisms such as phosphorylation and is involved in carbon partitioning [18]. Moreover, SUS catalyzes the reversible conversion of sucrose, serving as a critical metabolic node in sucrose metabolism, UDP-Glc supply, and cell wall/starch biosynthesis. The results of previous studies have demonstrated that reduced SUS activity impairs cellulose and starch synthesis, whereas overexpression of SUS increases their contents [19]. Collectively, these findings suggest the potential impact of GT4s on sink organ development. Furthermore, the authors of a recent study systematically identified the soybean sucrose phosphate synthase (*GmSPS*) gene family and analyzed the responses of its members to cold stress [20]. Despite the existing research on selected GT4 members, there remains a lack of systematic identification and characterization of the entire large *GmGT4* gene family in soybean.

In this study, we utilized the amino acid sequence information of GT4s from CAZy to construct a hidden Markov model for the systematic and comprehensive identification of these sequences within the soybean genome. We analyzed the characteristics of these members at the genomic, spatiotemporal transcriptomic, and protein structural levels. Furthermore, utilizing EMS-induced mutants, we identified a candidate gene regulating soybean seed weight. Through this work, we aim to provide a theoretical foundation for the functional analysis and mechanistic research of the soybean GT4 gene family.

## 2. Materials and Methods

### 2.1. Identification of GT4 Family Members

To identify members of the GT4 family within the soybean genome, we first retrieved the protein sequences of Arabidopsis GT4 family members from the CAZy database (CAZy, http://www.cazy.org/) (accessed on 23 July 2024) and downloaded the corresponding amino acid sequences from the NCBI database (https://www.ncbi.nlm.nih.gov) (accessed on 23 July 2024). After manually eliminating redundant sequences, we employed the hmmbuild module of the HMMER software package (v3.3.2) [21] to construct a hidden Markov model (HMM) for the GT4 family based on the non-redundant sequences. We then utilized hmmsearch to query the protein database of the soybean reference genome *G*. *max* Wm82.a4.v1 (Phytozome: ID 508; NCBI Taxonomy ID: 3847), setting the *e*-value threshold at 1 × 10^−6^. The initially obtained candidate sequences were subsequently verified through the NCBI WebCD database to ascertain the presence of typical GT-related protein conserved domains, leading to the identification of soybean GT4 family members (GmGT4s). The location information for the identified GmGT4-related genes was extracted from the genome annotation files provided by the JGI database and visualized using the Gene Location Visualizer (Advanced) function available in TBtools (v1.113) [22].

### 2.2. Phylogenetic Analysis of GmGT4 and AtGT4

Multiple sequence alignments of the protein sequences associated with AtGT4s and GmGT4s were conducted using the ClustalW program within MEGA11. The maximum likelihood phylogenetic tree was constructed using the tree construction tool provided by MEGA11, with a Bootstrap value set at 1000. The resulting nwk tree file was imported into iTOL (https://itol.embl.de/) (accessed on 18 October 2025) for visualization and enhancement. The branches corresponding to AtGT4s in the phylogenetic tree were utilized for the grouping and identification mapping of *GmGT4s*.

### 2.3. Analysis of Gene Structure, Conserved Motifs, and Protein Domains

The conserved motifs were predicted using the local tool MEME Suite (v5.5.0) [23], provided by MEME (http://meme-suite.org/) (accessed on 18 October 2025), with parameters set to retrieve three motifs and a specified amino acid sequence length of 15. The NCBI WebCD search tool (https://www.ncbi.nlm.nih.gov/Structure/bwrpsb/bwrpsb.cgi) (accessed on 18 October 2025) was utilized to identify conserved protein structural domains. The *G*. *max* (Wm82.a4.v1) genome annotation file, downloaded from the JGI database, was employed to illustrate gene structure information, while the sequence data for the EMS mutagenic material were obtained from the soybean database (http://isoybean.org/) (accessed on 18 October 2025) [24]. To obtain three-dimensional structural information of proteins, modeling and visualization were conducted using the SWISS-MODEL database [25], with wild-type and mutant protein sequences serving as input.

### 2.4. Collinearity Analysis and Selection Pressure Calculation

Intra-species collinearity analysis and selection pressure calculation are conducted in three primary steps. First, *G*. *max* (Wm 82 a4.v1) is obtained from the JGI database. The CDS sequence information is then subjected to multiple sequence alignment using the local BLAST tool (https://blast.ncbi.nlm.nih.gov/Blast.cgi) (accessed on 18 October 2025), *e*-value 1 × 10^−5^. Next, linear analysis is performed with MCScanX (https://github.com/wyp1125/MCScanX) (accessed on 18 October 2025) [26]. Collinear regions were identified using a match score ≥ 50, a minimum of 5 matching genes, and a maximum of 25 gaps. Finally, the Advanced Circos function provided by TBtools is utilized for visualization, emphasizing the genes associated with GmGT4s. For selection pressure analysis, direct homologous genes of the soybean target gene are identified using the common bean reference genome *Phaseolus vulgaris* v2.1. Subsequently, the MAFFT tool (v7.525) is employed for comparison and gap removal. The Ka and Ks values for each direct gene pair are calculated using KaKs_Calculator (v 2.0) [27] to assess selection pressure.

### 2.5. Analysis of Organizational Expression Patterns and Spatiotemporal Expression Patterns

Expression levels of soybean GmGT4s-related genes during the growth and development of various tissues and organs were obtained from the National Center for Biological Information Soybean Genomics Database (https://ngdc.cncb.ac.cn/soyomics/index) (accessed on 19 October 2025), analyzed using the Circlize R package (https://jokergoo.github.io/circlize/) (accessed on 19 October 2025), and visualized with ComplexHeatmap (https://github.com/jokergoo/ComplexHeatmap) (accessed on 19 October 2025).

Single-cell and spatial transcriptomic subsets of soybean seeds during the early maturation stage were obtained from a public dataset (GEO: GSE270392) [28]. Utilizing the data filtering, cell clustering, and annotation information provided in the original dataset, the single-cell expression UMAP and spatial expression profile of the target gene were visualized using the Seurat R package (version 5.0) [29]. To create the cell type expression heatmap, Seurat was employed to extract the average expression level of the target gene across each cell type, followed by the use of the Heatmap package to visualize the standardized cell type expression. Furthermore, cell type marker genes were identified using the FindAllMarkers function with parameters set to min.pct = 0.25 and logfc.threshold = 0.25.

### 2.6. Construction of the Co-Expression Gene Network

The expression matrix was extracted from the single-cell transcriptomic dataset using the Seurat R package (v.5.5.0). Spearman correlation coefficients between the target gene and all genome-wide genes were calculated. *p*-values were estimated using the t-distribution approximation method, and multiple testing correction was performed using the Benjamini–Hochberg (BH) method. Gene pairs with an absolute correlation coefficient > 0.1 and an FDR < 0.01 were ultimately selected to construct the co-expression network.

### 2.7. GO Enrichment Analysis

The GO Term Enrichment Tool function available through the Soybase database (https://soybase.org/) (accessed on 19 October 2025) was utilized for the enrichment analysis of Gene Ontology (GO) terms, specifically focusing on biological pathways (BP) with a *p*.adjust threshold of less than 0.05. The resulting enrichment terms were visualized using the R package ggplot2.

### 2.8. Haplotype Analysis and Domestication Selection Analysis

Based on the positional information of candidate gene mutations extracted from group heavy sequencing data, haploid type analysis was conducted using the R package geneHapR (version 1.2.5) [30] (https://gitee.com/zhangrenl/genehapr) (accessed on 19 October 2025). The reference genome utilized was the *G*. *max* (Wm82.a4.v1) version from the JGI database. This analysis retained only the variation information from the CDS region and excluded heterozygous sites. The 100-seed weight phenotypes corresponding to each haplotype were employed for haplotype-phenotypic preliminary association analysis.

Genetic diversity analysis (π) and the genetic differentiation index (Fst) were computed using the Vcftools (version 0.1.16) [31]. Nucleotide diversity (π) was calculated using a sliding window approach with a window size of 100 kb and a step size of 20 kb. The genetic differentiation between populations was estimated using the Weir & Cockerham method, also employing a sliding window of 100 kb with a 20 kb step. Visualization of the results was conducted using ggplot2.

### 2.9. qRT-PCR Analysis

At different developmental stages (R4–R7) of soybean (Williams 82), the middle seeds from three-seeded pods with normal growth conditions were selected and flash-frozen. Each developmental stage included at least six biological replicates (independent plants). Total RNA was extracted using a QIAGEN RNA extraction kit (QIAGEN, Hilden, Germany) according to the manufacturer’s instructions, and its integrity was verified by 1% agarose gel electrophoresis.

Subsequently, total RNA was reverse transcribed into complementary DNA (cDNA) utilizing HiScript II Q RT SuperMix (Vazyme, Nanjing, China) for qPCR (+gDNA wiper). Then, quantitative real-time PCR (qRT-PCR) amplification was conducted on the StepOne Plus™ system with SYBR qPCR Master Mix (Vazyme, Nanjing, China). Glyma.04G215900 (Actin) was used as an internal reference gene, and the relative expression levels of the target genes were calculated using the 2^−ΔΔCt^ method. Primer sequences are provided in Table S1.

### 2.10. Soybean Cultivation and 100-Seed Weight Investigation

All soybean materials utilized for phenotypic analysis were planted at the Agricultural Experiment Station of Shandong Agricultural University in Tai’an, China (36.16° N, 117.16° E). The materials employed for comparing seed size were cultivated under uniform conditions, with a plant spacing of 10 cm and a row spacing of 50 cm in 3 m × 5 m plots, along with three replicated plots.

The EMS materials were obtained from Nanjing Agricultural University in 2023. All mutant seeds used for measuring seed-related parameters were self-pollinated for two generations. They were subsequently used only after genotyping confirmed that the candidate gene loci were homozygous mutants. Genotyping was performed using KASP markers. The KASP primers were designed using the online tool provided by Goodbtk (Guangzhou, China) (https://tools.goodbtk.com/SNPPrimer) (accessed on 30 August 2026). The genotyping assays were carried out on a StepOne Plus™ system using the Goodbtk Genotyping Kit (Cat. No. FLU-ARMS for KASP 2×PCR Mix V5F) (Guangzhou, China) according to the manufacturer’s instructions.

Seed length, width, and hundred-grain weight were measured using a Tuopu Cloud-agriculture soybean hundred-grain weight phenotyping platform. All seeds used for measurement were fully mature and harvested, then thoroughly dried to a moisture content of less than 12.5%. For each family line, at least three independent plants served as biological replicates for evaluation. Each replicate was weighed independently, with no less than 100 seeds per weighing. The hundred-grain weight was calculated based on the mean value of three independent weighings.

### 2.11. Statistical Analysis

Statistical analyses and data visualization were performed using Python (v3.12) and R (v4.4). For error analysis, all continuous variables are presented as the mean ± standard deviation (SD). For comparative analyses, pairwise comparisons were conducted using a two-tailed Student’s *t*-test, while comparisons among three or more groups were evaluated using a one-way analysis of variance (ANOVA) followed by Tukey’s honestly significant difference (HSD) post hoc test. A *p*-value < 0.05 was considered statistically significant.

## 3. Results

### 3.1. Identity Recognition and Grouping of Soybean GT4s

**Figure 1:**
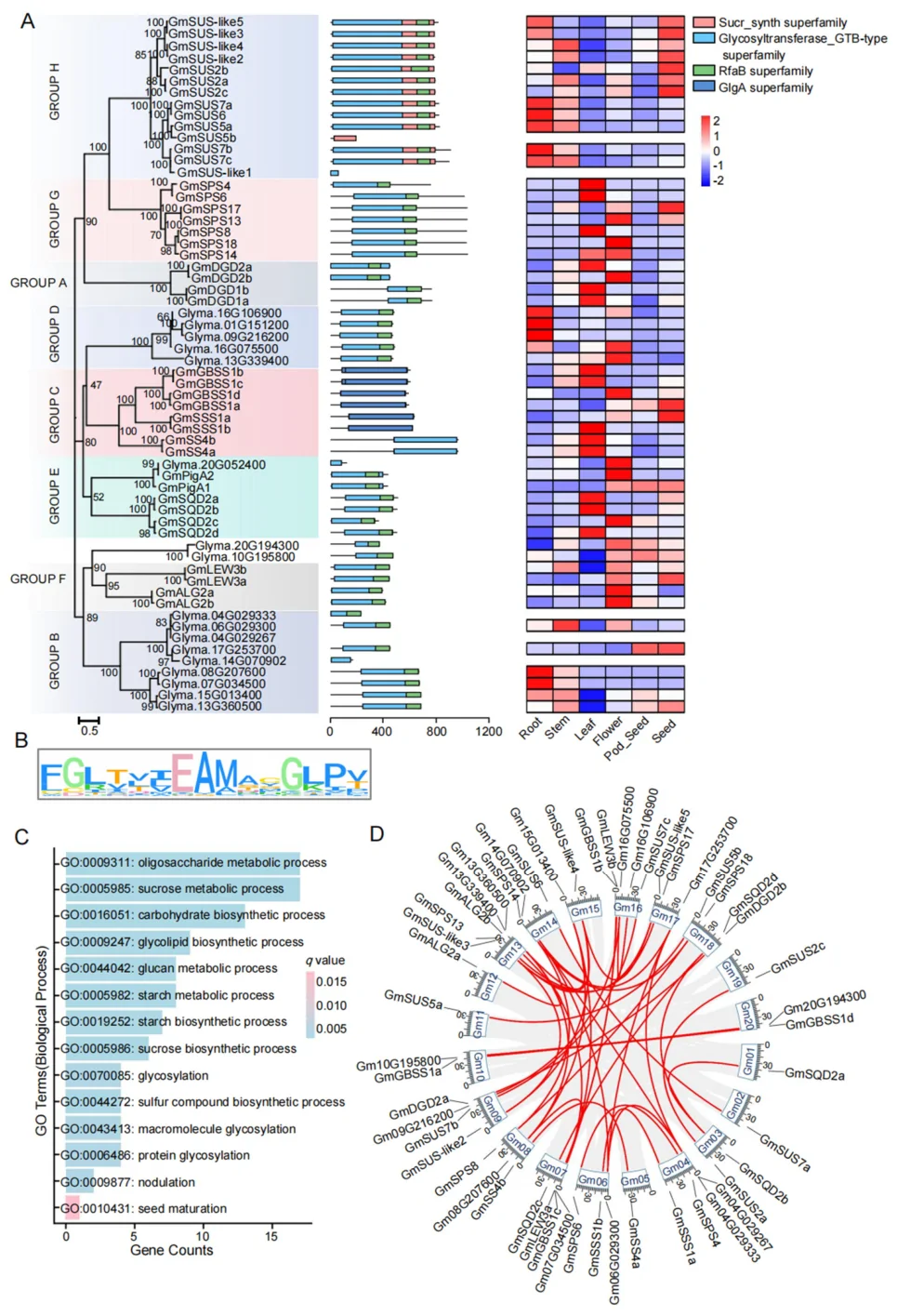
Analysis of the GmGT4 gene family. (**A**) Phylogenetic tree, protein domains, and tissue-specific expression patterns of the GT4 family in soybean. (**B**) Characteristic motifs of the GmGT4 gene family. (**C**) GO enrichment analysis of GmGT4s (Biological Process) with a q-value threshold of <0.05, including removal of redundant closely related terms. (**D**) Syntenic relationships among GmGT4 family-related genes. Red lines indicate syntenic relationships among GT4 family members, while gray lines represent those of other genes.

Utilizing the amino acid sequence data of Arabidopsis GT4s from the CAZy database, we constructed a hidden Markov model (HMM) for the GT4 family and subsequently identified GT4 family members in soybeans. Screening was performed based on *e*-values and conserved protein domains, resulting in the identification of 60 GmGT4 genes encoding 162 protein isoforms (Table S2). A comparative analysis of the GT4 families in *A. thaliana* and soybean was conducted, leading to the construction of a phylogenetic tree (Figure S1). Excluding two members that could not be assigned to any group, the remaining 58 GmGT4s were categorized into eight distinct groups (Figure 1A). The number of members within these groups varies from 4 to 14. Notably, Group H, associated with sucrose synthase, contains the highest number of GT4s, whereas Group A, linked to glycolipid synthesis, and Group F, related to N-glycosylation, each comprise only four members.

A comparative analysis of the structural features of GmGT4s was conducted, combined with the integration and visualization of the phylogenetic tree, motif, and gene structure (Figure S2). The results indicated that the majority of *GmGT4s* (*n* = 55) contained introns, with only five genes lacking introns, all of which were classified within Group D. The MEME tool was subsequently employed to predict three highly conserved motifs in GT4s, revealing that members of the same group exhibited similar motif compositions. By further integrating these motifs, we delineated the characteristic motifs of the GT4 family (Figure 1B and Figure S3). Additionally, an analysis of the protein conserved domains using the NCBI CDD database demonstrated that the CDD structures of most members were analogous, with a high degree of consistency observed among members within the same group (Figure 1A).

### 3.2. Functions and Tissue Expression Patterns of GmGT4-Related Genes

Utilizing gene function annotation data from the DAVID database [32], we compiled the functional information of *GT4* members (Table S3). Notably, the sequence-based classification exhibited a strong correlation with gene function. Group A encodes galactoglyceryl diester (DGD) synthase. Group C members primarily facilitate starch synthesis. Groups E and F encode enzymes associated with sulfoquinovosyl transferase (SQD) and protein glycosylation, respectively. Groups G and H contain a substantial number of members, all of which are involved in sucrose metabolism. Group G is responsible for sucrose anabolism, while Group H reversibly catalyzes both the synthesis and degradation of sucrose. The functions of the members in Groups B and D remain unclear.

To gain deeper insights into the biological pathways associated with *GT4s*, we performed GO enrichment analysis [33] on the identified genes, revealing significant enrichment of 14 GO terms (*q*-value < 0.05) (Figure 1C; Table S4). Notably, in addition to pathways linked to glucose metabolism (such as GO:0005985 and GO:0009247) and glycosylation modification (GO:0006486), pathways associated with nodulation (GO:0009877) and seed maturation (GO:0010431) were also enriched.

Functional differentiation leads to variations in expression patterns. To comprehend the roles of *GT4s* across different growth stages and tissue locations, we constructed a heat map illustrating the tissue-specific expression profiles of the *GT4* gene family (Figure 1A). As anticipated, functionally related GT4s within the same cluster displayed similar expression patterns. For example, members of the GmSUS family encoding sucrose synthase showed relatively high expression levels in organs such as roots, stems, and seeds, whereas Group G members encoding GmSPS were predominantly highly expressed in leaves and flowers. Notably, the E group members responsible for sulfoquinovosyl transferase (SQD) demonstrated a diverse expression pattern, potentially underpinning the multifaceted functions of SQD.

### 3.3. Chromosomal Distribution and Replication Phenomena of GT4s

All *GmGT4* genes were distributed across 20 soybean chromosomes, demonstrating their extensive presence. Each chromosome harbors *GT4* genes, with chromosome 13 exhibiting the highest number (*n* = 6). In contrast, four chromosomes contain only a single gene (Figure S4). Overall, *GmGT4* genes exhibited no clustering phenomenon.

Intraspecific collinearity analysis revealed that, of the 60 GmGT4s, 52 exhibited collinear genes, with some containing multiple collinear genes. Notably, the four genes in Group B—*Glyma.07G034500*, *Glyma.08G207600*, *Glyma.13G360500*, and *Glyma.15G013400*—demonstrated a collinear relationship (Figure 1D), providing direct evidence of multiple whole-genome duplications. In contrast, eight genes displayed single-copy characteristics, with no collinear genes identified. Furthermore, no tandem repeat events were detected among these genes.

### 3.4. GmGT4s Is Subject to Varying Degrees of Selection Pressure

The unique whole-genome replication event in soybeans has conferred greater evolutionary resilience and extensive adaptive potential to the soybean genome. To assess the impact of genome-wide replication on members of the *GT4* family, we examined the selection pressure on *GmGT4s* through direct homology mapping with *GmGT4s* in common beans (*P. vulgaris* L.) (Figure S5). Notably, as illustrated in Table S5, all members displayed characteristics of purifying selection. Eight genes experienced stronger selection pressure (*Ka/Ks* < 0.1), including four that encoded sucrose synthase and two that encoded granule-bound starch synthase. At the group level, members within the same group also exhibited varying selection pressures. Starch synthase and soluble starch synthase, both classified under Group C, faced relatively relaxed selection pressures in comparison to *GmGBSS*.

### 3.5. Single-Cell and Spatial Expression Patterns of GmGT4s

**Figure 2:**
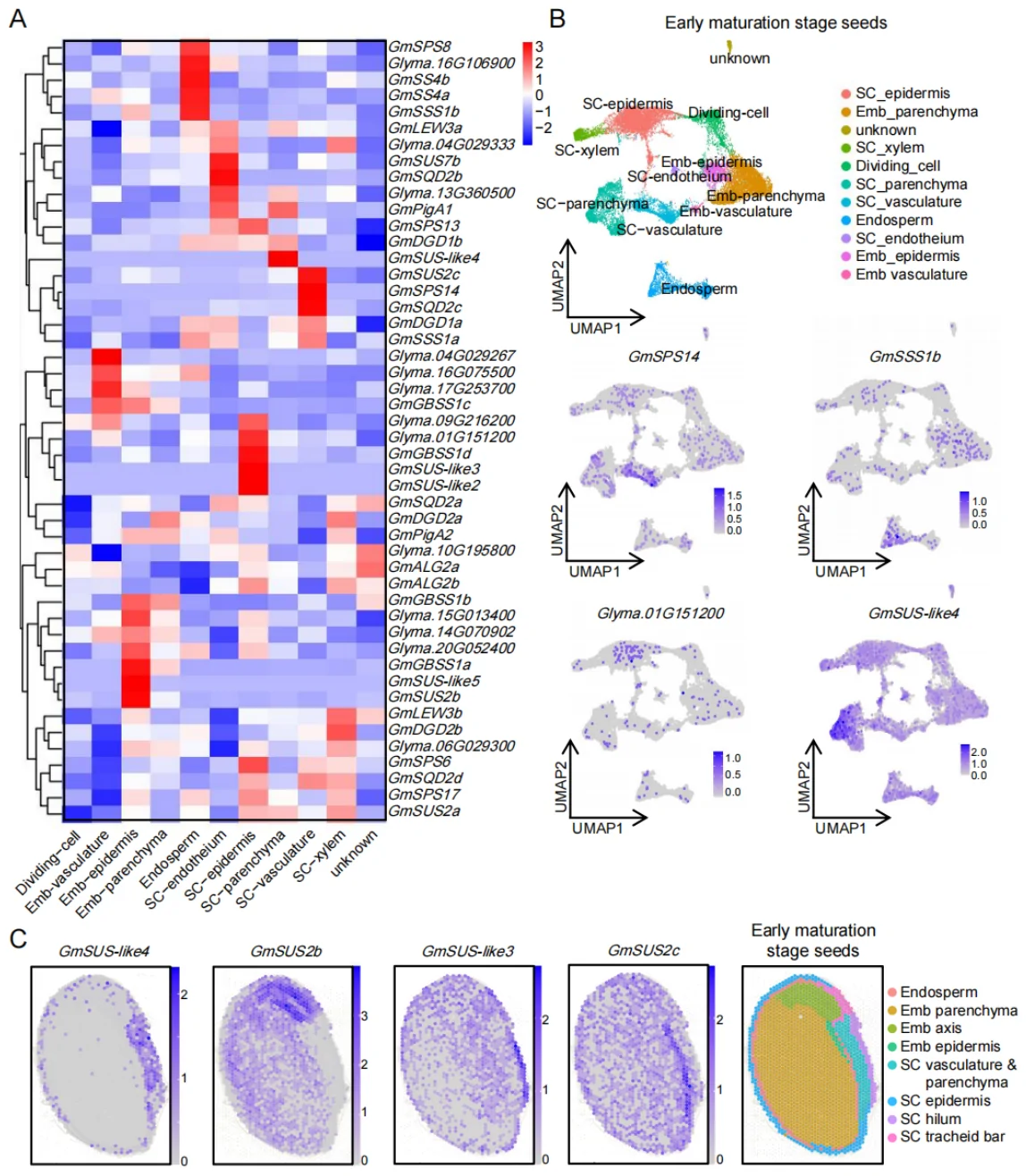
Single-cell and spatial expression patterns of the GmGT4s. (**A**,**B**) Single-cell expression heatmap of the GmGT4s gene and UMAP visualization of selected genes. The data were derived from single-nucleus RNA sequencing (snRNA-seq) of soybean seeds at the early developmental stage. For the heatmap, expression intensity was prioritized over the number of expressing cells, and data were normalized per gene. For the UMAP, the visualization was constructed based on the normalized expression profiles. (**C**) Spatial expression patterns of GmGT4s in soybean seeds at the early developmental stage.

Most members of the GmGT4 family are involved in the allocation of sucrose, which has sparked our interest in their role in seed development. By publicly sharing the single-cell and spatial transcriptome datasets of soybean seed development [28], we compiled and generated the single-cell (Figure 2A,B) and spatial expression profiles (Figure 2C) of *GmGT4* family members in seed during the early maturation stage. Unlike tissue expression patterns, the same group of members exhibited distinct cell type-specific expressions. For example, *GmSPS8*, which encodes sucrose phosphate synthase (SPS), was predominantly expressed in the endosperm, whereas *GmSPS14* exhibited high expression in the vascular cells of the seed coat. In conjunction with the spatial expression map, we conducted a comprehensive comparison of the expression characteristics of several key members encoding sucrose synthase (SUS). Notably, *GmSUS2b* was identified in the embryonic epidermis and embryonic axis, *GmSUS2c* was found in the vascular cells of the germplasm, and *GmSUS-like3* and *GmSUS-like4* displayed specificity in the seed coat epidermis and parenchyma cells, respectively. *GmSUS2a* did not exhibit distinct cell type expression characteristics. Furthermore, we identified six cell type marker genes within the GmGT4 family (Table S6, Figure S6), including four specific markers: GmSUS-like3 and GmSUS-like4 for seed coat epidermal cells and GmSUS-like5 and GmGBSS1c for embryo epidermal cells.

### 3.6. Identification of Candidate GmGT4 Genes Potentially Affecting Seed Development Using EMS Materials

**Figure 3:**
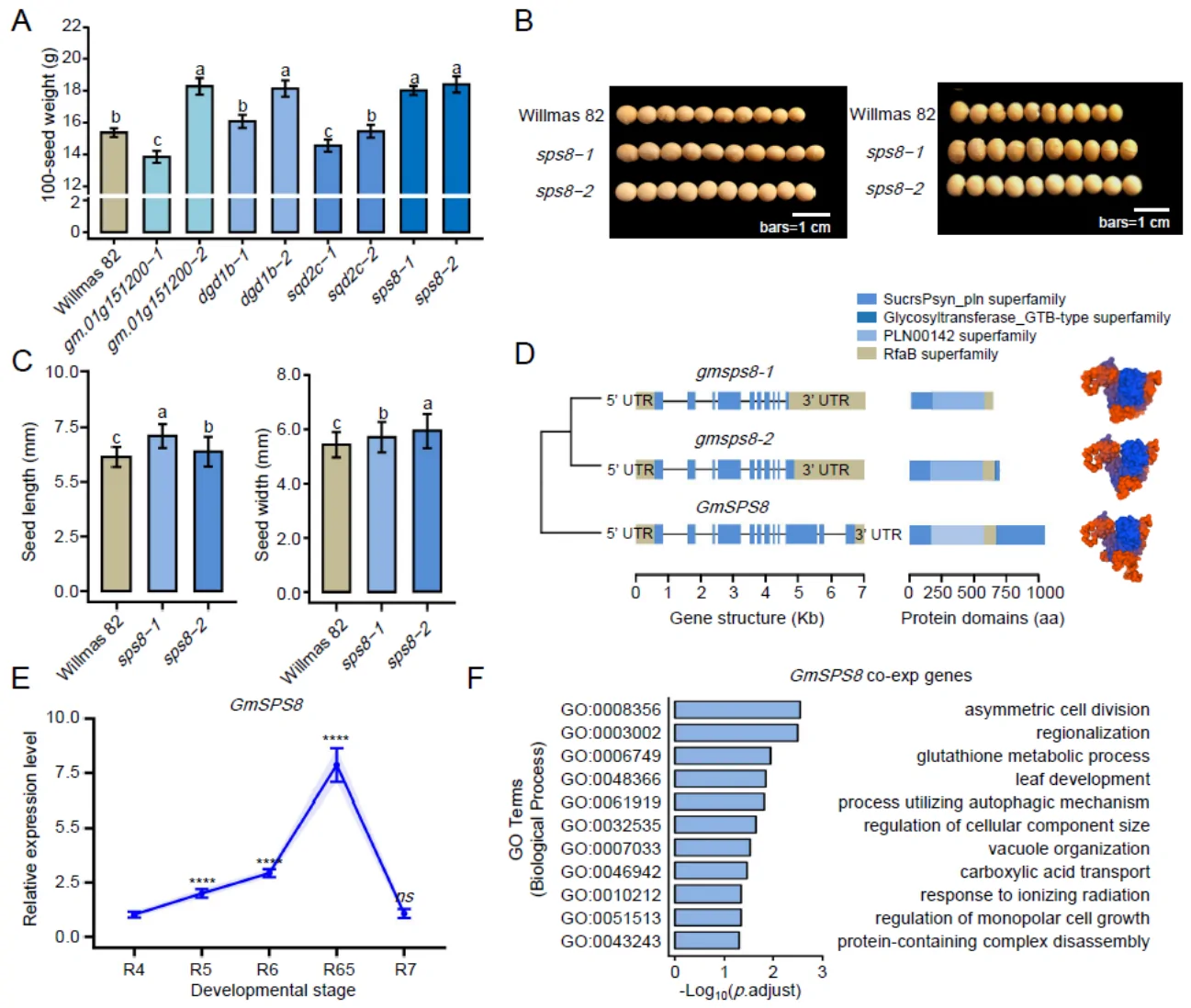
GmSPS8 is a candidate gene associated with soybean seed size traits. (**A**) Hundred-seed weight phenotypes of EMS-induced premature termination mutants of selected GmGT4s genes, with each mutant line comprising at least three biological replicates. Error bars indicate the standard deviation (SD). (**B**,**C**) Phenotypes of the *GmSPS8* premature termination EMS mutants (seed length and seed width). Error bars indicate the standard deviation (SD). (**D**) Gene structure and protein features of the *GmSPS8* premature termination EMS mutant, together with its protein conformation. The protein conformation was generated using SWISS-MODEL. (**E**) Temporal expression pattern of *GmSPS8* at different seed developmental stages. Error bars indicate the standard deviation (SD). (**F**) GO enrichment analysis of genes co-expressed with GmSPS8 based on single-cell datasets (*p*.adjust < 0.05). Statistical significance is indicated as follows: ****, *p* < 0.0001; ns, not significant (*p* > 0.05). Different letters (a, b, c) indicate significant differences (*p* < 0.05), while groups sharing the same letter are not significantly different.

Single-cell and spatial expression profiles have elucidated the diverse functions of GT4s in seed development, while EMS mutagenesis facilitated the discovery of candidate genes underlying these biological functions. By screening 221 EMS mutagenic materials for GT4S-related mutants, we identified four pairs of homozygous termination mutant materials corresponding to GT4s members and performed a statistical analysis of their seed sizes (Table S7; Figure 3A). Although certain mutant lines of *Glyma.01G151200*, *GmDGD1b*, and *GmSQD2c* exhibited significantly different 100-seed weight phenotypes, both mutant systems of *GmSPS8* displayed increased 100-seed weight. Further analysis indicated that the seed length and width of the two *GmSPS8* mutant lines were significantly enhanced (Figure 3B,C), with *gmsps8-1* seeds being longer and *gmsps8-2* seeds being wider. We determined the genotypes of these two mutant lines (Tables S8 and S9; Figure S7) and analyzed their gene and protein structures (Figure 3D). We found that both retained the complete PLN00142 superfamily domain, although the functional domain of the SucrsPsyn_pln superfamily was disrupted.

To enhance our understanding of GmSPS8’s role in seed development, we first analyzed its temporal expression patterns across various stages of seed development (Figure 3E). The results indicated that *GmSPS8* expression levels gradually increased during the early stages of seed development, peaking around the R6–R6.5 stage (spanning from the granulation stage to the early maturity stage) before declining during the R6.5–R7 stage (maturity stage). Subsequently, we mined the co-expressed genes of GmSPS8 using the aforementioned single-cell transcriptomic dataset (Figure S8; Table S10) and performed GO enrichment analysis. Interestingly, these co-expressed genes were significantly enriched in asymmetric cell division (GO:0008356) and regulation of cellular component size (GO:0032535) (Figure 3F).

### 3.7. Natural Variations in GmSPS8 Potentially Affect Seed Size

**Figure 4:**
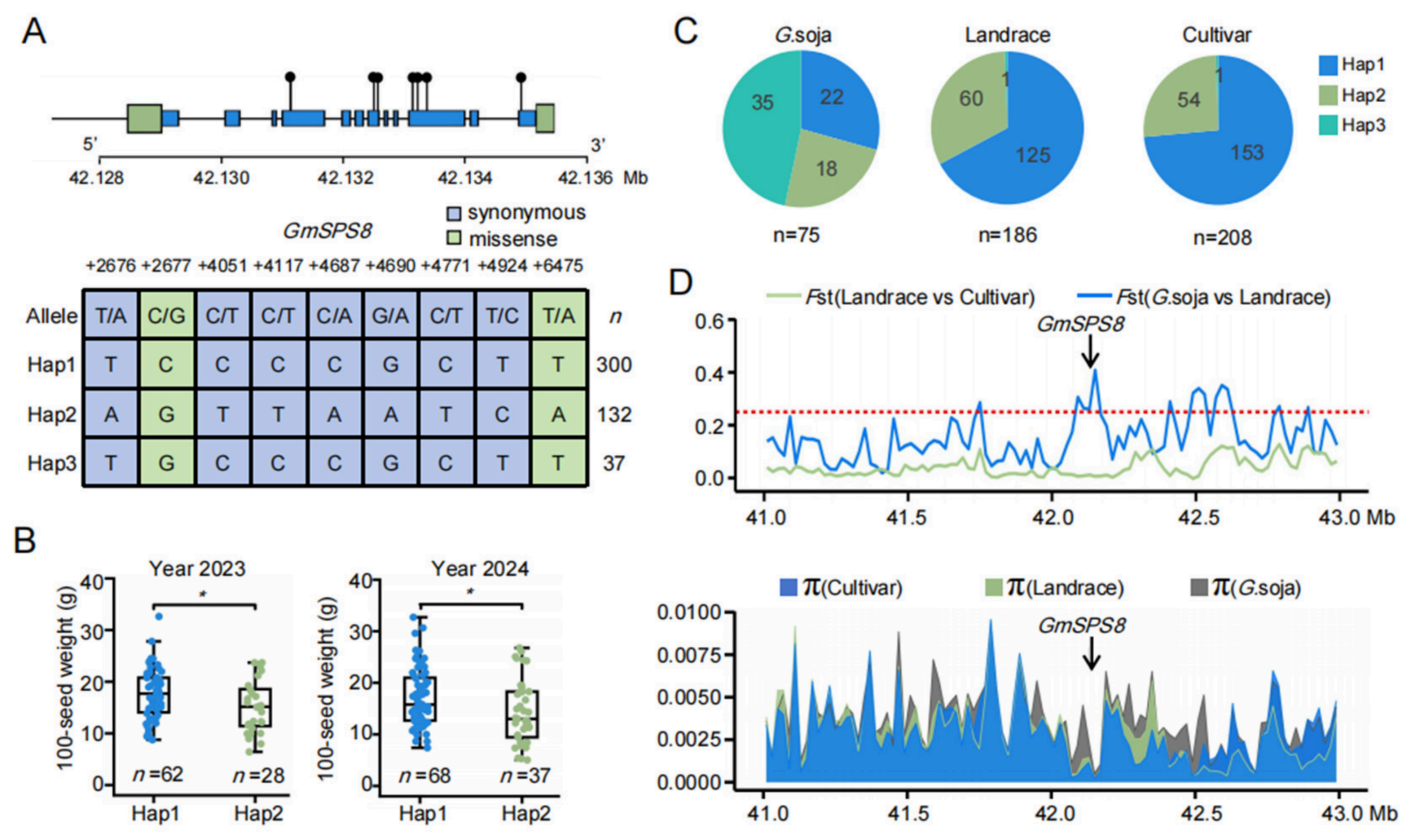
Preliminary haplotype analysis of *GmSPS8*. (**A**) Haplotype analysis of *GmSPS8* based on 559 natural germplasm resources: heterozygotes were removed, and haplotypes with a frequency exceeding 5% were retained. The letters A, T, C, and G represent the four nucleotide bases (adenine, thymine, cytosine, and guanine). (**B**) Hundred-seed weight of different haplotype-specific landrace germplasm in 2023 and 2024. Error bars indicate the standard deviation (SD). (**C**) Distribution of different haplotypes across germplasm subpopulations. (**D**) Analysis of FST (fixation index) and π (nucleotide diversity) of *GmSPS8* across 559 natural germplasm accessions. The red threshold line indicates the top 5% detection threshold at the genome-wide level. Statistical significance is indicated as follows: *, *p* < 0.05.

The identification of EMS mutants suggested that GmSPS8 potentially affects seed size, prompting our interest in whether its variation under natural conditions affects seed phenotype. Utilizing whole-genome resequencing data from 559 soybean germplasm resources [34], we examined all natural variations within the *GmSPS8* exon region, which included seven synonymous mutations and two missense mutation SNPs. Based on the principle of retaining haplotypes with a frequency greater than 5%, nine variant sites classified 469 out of 559 accessions into three distinct haplotypes, with 300 classified as haplotype 1 and 37 as haplotype 3 (Figure 4A and Figure S9; Table S11). To investigate the potential impact of these natural variations on seed size, we selected a subset of landrace subgroups that exhibited relatively minor genetic background differences among the 469 germplasms for haplotype and phenotype preliminary association analysis. The results indicate (Table S12, Figure 4B) that the seed size of haplotype 1 is significantly larger than that of haplotype 2.

### 3.8. Localization of GmSPS8 Within a Domestication Selection Region

In previous studies [35], researchers have identified seed size as a significant domestication trait in soybeans, with genes regulating this trait often undergoing artificial selection throughout the extensive process of domestication and improvement. Consequently, we analyzed the distribution of *GmSPS8* haplotypes across cultivar, landrace and wild variety (*G. soja*) subgroups (Table S12, Figure 4C). Notably, the proportion of haplotype 1 in landrace and cultivar is significantly higher than that observed in wild varieties, whereas haplotype 3 predominantly occurs in wild variety subgroups and is almost absent in cultivar and landrace. We then calculated the genetic differentiation index and genetic diversity among the different subgroups (Figure 4D). As anticipated, *GmSPS8* is located within a domestication selection region.

## 4. Discussion

In this study, we systematically analyzed the gene characteristics, protein domains, and functional divergence of the soybean GT4 family. Furthermore, we investigated the expression profiles of each family member using bulk, single-cell and spatiotemporal transcriptomic data. Utilizing EMS materials, we identified *GmSPS8* as a candidate gene potentially regulating seed size. Subsequent analyses of haplotypes and domestication selection parameters in a natural population revealed that *GmSPS8* resides within a domestication selection region. These findings advance our understanding of the glycosyltransferase family in soybean.

### 4.1. Complex Features and Functions of the GmGT4 Family

The glycosyltransferase superfamily is extensive and intricate [4], encompassing nearly all biological categories and playing crucial roles in various biological processes [36]. Notably, while these members exhibit relatively conserved secondary and tertiary structures, they display considerable sequence variation [1], leading to their classification into distinct families. As the second-largest glycosyltransferase family, the characteristics and physiological functions of some soybean GT4 members have attracted considerable attention [18,19]. The authors of previous studies have also identified soybean sucrose phosphate synthase (GmSPS) members and analyzed their responses to cold stress [20]; however, the entire large GT4 family has not yet been fully identified and classified. In this study, a hidden Markov model was developed utilizing the sequence information from the *A. thaliana* GT4 family in the CAZy database, resulting in the identification of 60 GmGT4 members. The genomic and transcriptional characteristics of these members were preliminarily analyzed to uncover commonalities.

Firstly, due to multiple genome-wide replication events in the soybean genome [37], most gene members exhibit complex structures. In contrast, members of group D display simpler gene architectures, with five lacking introns entirely. These members perform various biological functions and possess a limited number of homologous copies within their genomes. Compared to members of other groups that exhibit functional redundancy, such as the starch synthase and sucrose-phosphate synthase families, group D members appear to maintain greater conservation under significant selection pressure. This perspective is further supported by the results of the *Ka/Ks* analysis. Regarding expression levels, distinct organizational expression patterns are observed among different groups of *GT4s*, while members within the same group demonstrate varying expression patterns across cell types. The characteristics of spatiotemporal expression often suggest functional redundancy and differentiation. For instance, *GmSS4a* and *GmSS4b*, both encoding starch synthase, exhibit highly consistent tissue and single-cell expression profiles. In contrast, the four members of *GBSS1s*, which also encode granule-binding starch synthase, display entirely different tissue and cell type expression patterns.

In contrast to GT1s, which primarily utilize UDP as a substrate [38], GT4s exhibit a greater diversity of substrates and products, in addition to more intricate functions. The results of Gene Ontology (GO) enrichment analysis for the *GT4* gene family further support this observation. *GmSUS-like3*, which encodes sucrose synthase, and *GmDGD2b*, which encodes galactosyl diacylglycerol synthase, have been annotated as being associated with rhizome symbiosis. In alfalfa, the inhibition of the antisense rhizome symbiosis of *MtSUS1* resulted in delayed rhizome development and reduced nitrogen fixation efficiency [39]. Additionally, the number of root nodules and nitrogen fixation activity in the *MtDGD1*-RNAi strain were significantly lower compared to those in the normal strain [40]. Moreover, *GmSUS2b*, which encodes sucrose synthase, has been annotated as influencing seed maturation, a finding that is also supported in *A. thaliana* [41]. In summary, the *GT4* gene family demonstrates complex structural and expression characteristics, thereby exhibiting diverse and variable biological functions.

### 4.2. GmSPS8 Serves as a Candidate Gene for the Regulation of Seed Size

Through screening of EMS materials, we identified GmSPS8, a member of the GmGT4 family, as a candidate gene potentially regulating seed size. Sucrose phosphate synthase (SPS) is a key enzyme in sucrose metabolism and has been extensively studied for its role in sucrose synthesis [42]. In soybean, the results of previous studies have demonstrated the relationship between sugar accumulation and SPS activity in seeds [43], and suggested that SPS may play an important role in responding to abiotic stresses [44]. More recently, a research group systematically identified GmSPS family members and comprehensively analyzed their sequence and expression characteristics, including their expression changes under cold stress; notably, they study specifically highlighted the response of *GmSPS8/18* to cold stress [20]. While the authors of previous studies have established GmSPSs as an important metabolic enzymes involved in various physiological processes during soybean growth and development, our findings suggest their potential function in regulating soybean seed development. Furthermore, the temporally differential expression patterns across different developmental stages and the spatial expression patterns during the early maturation stage imply their spatiotemporal functional specificity.

GmSPS8 contains two protein domains: the PLN00142 superfamily domain and the SucrsPsyn_pln superfamily domain. The former is a characteristic domain of sucrose synthase (SUS), which mediates the conversion of fructose to sucrose using UDP-glucose [45]. SUS also reversibly catalyzes sucrose degradation, thereby promoting the synthesis of various polysaccharides, including starch and cellulose [46,47]. Notably, all of the EMS mutants identified in our study harbored mutations that disrupted the SucrsPsyn_pln superfamily domain while retaining the PLN00142 superfamily domain. This structural alteration may have potentially influenced soybean seed size.

### 4.3. GmSPS8 Potentially Experienced Artificial Selection During Domestication

By examining the natural variations in *GmSPS8*, we determined that this gene may have played a role in the domestication of soybeans. Throughout the transition from wild to landrace varieties, the genetic diversity of *GmSPS8* experienced a rapid decline, resulting in the near extinction of haplotype 3, which comprised half of the wild varieties, in landrace and cultivar. The authors of previous research has indicated that soybean seed size was a trait directly selected during the domestication of soybeans [35]. As *GmSPS8* resides within a domestication selection region and its haplotypes are significantly associated with seed size, we propose that this gene may have undergone artificial selection. Despite the considerable genetic variability among wild variety materials, the association analysis linking their haplotypes to seed size supports our hypothesis (Table S12, Figure S10). Notably, during the improvement process, the frequency of haplotype 1, which is associated with larger seeds, continued to rise and was further favored.

## 5. Conclusions

In this study, we comprehensively identified 60 *GT4s* in the soybean genome and analyzed their structural and tissue expression characteristics. By integrating single-cell and spatial transcriptomic data of soybean seeds during the early maturation stage, we further elucidated the unique spatiotemporal expression patterns of these GT4 members. Utilizing EMS materials, we identified *GmSPS8* as a candidate gene potentially regulating seed size. Furthermore, analyses of natural variations and haplotypes suggest that *GmSPS8* may have undergone artificial selection. Our findings provide a valuable foundation for understanding GT4 family functions and identify a potential candidate gene for genetic improvement of seed size in soybean.

## Data Availability

The original data presented in the study are openly available in the Genome Sequence Archive (GSA) database of the BIG Data Center at PRJCA039917.

## Supplementary Materials

The following supporting information can be downloaded at https://www.mdpi.com/article/10.3390/cimb48090918/s1.
