# Supplementary material for: Genome-Wide Identification of the Glycosyltransferase 4 Family and Characterization of GmSPS8 Associated with Seed Weight in Soybean
Source: Curr Issues Mol Biol. 2026 Sep 8;48(9):918. doi: 10.3390/cimb48090918 (PMC13605089; doi:10.3390/cimb48090918)
Supplement: Supplementary file 1 [file cimb-48-00918-s001.zip › Supplementary Figures.pdf]

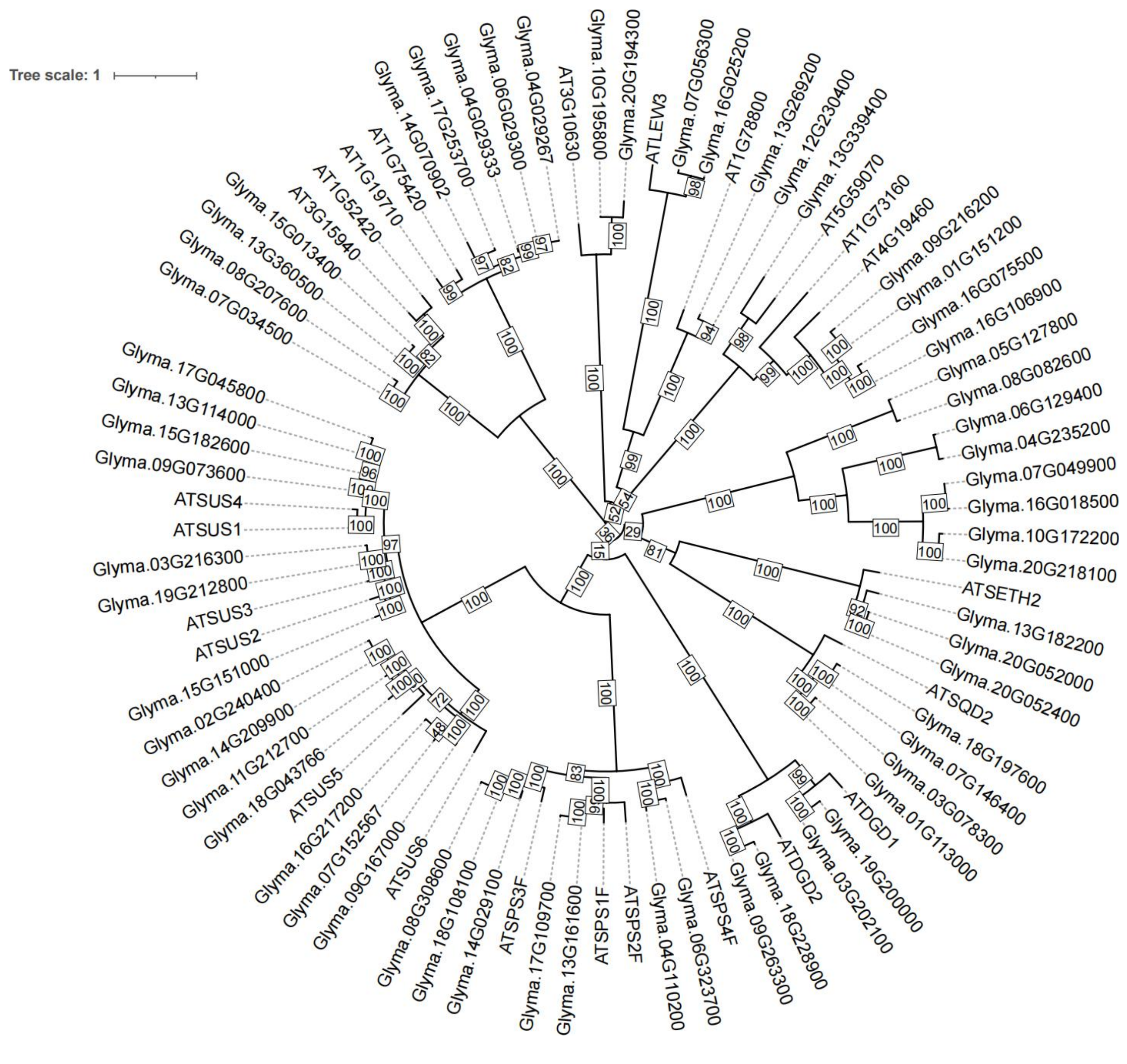


**Figure S1. Phylogenetic analysis of GT4 family members.** Phylogenetic analysis of GT4 family members. Phylogenetic tree of the GT4 family in soybean and Arabidopsis thaliana. Constructed using the maximum likelihood (ML) method in MEGA11 with Bootstrap replication set to 1000 times, with all other parameters maintained at default values.

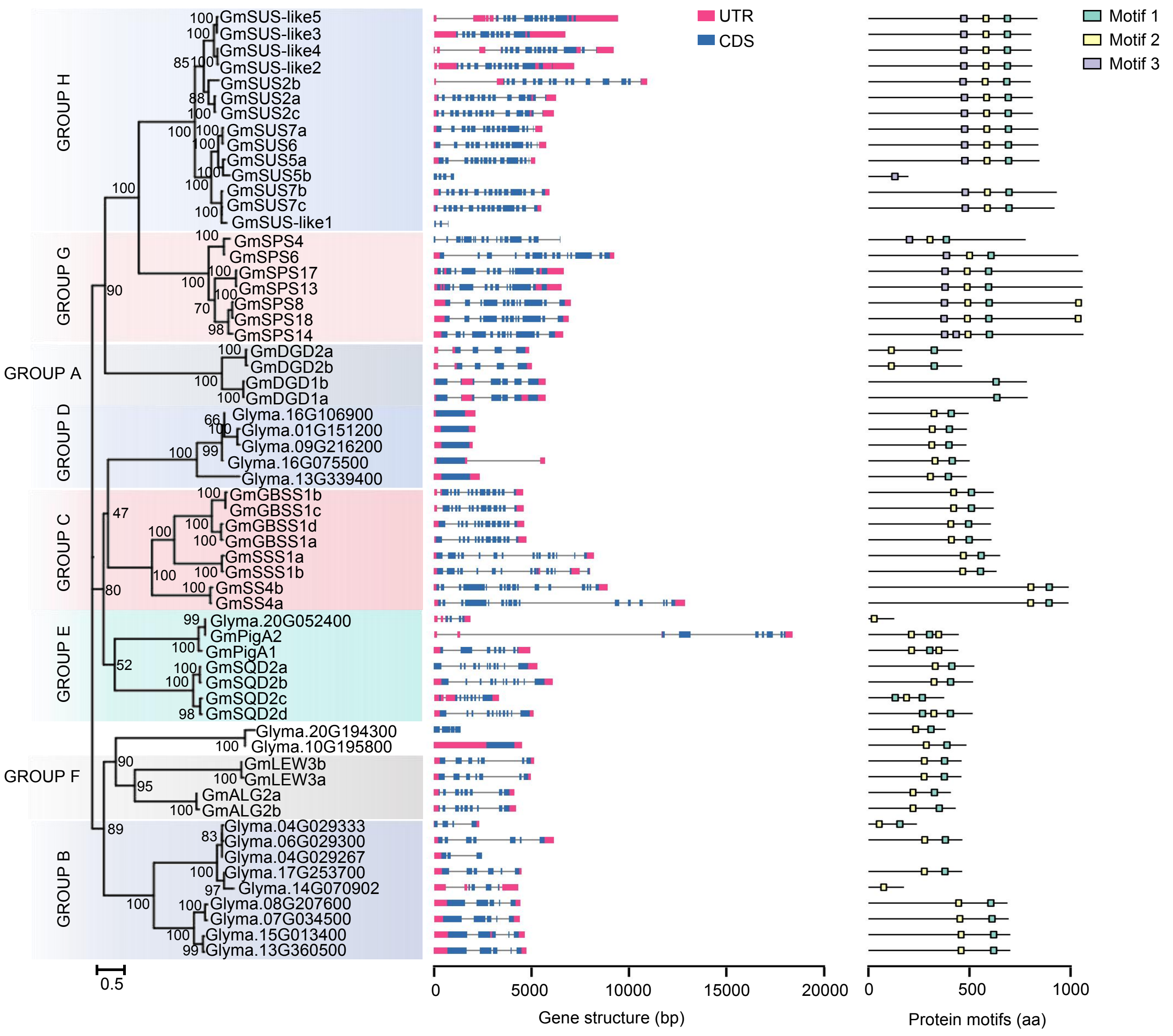


**Figure S2. Characterization of gene structures and conserved amino acid motifs in the 60 GmGT4 gene family members.**

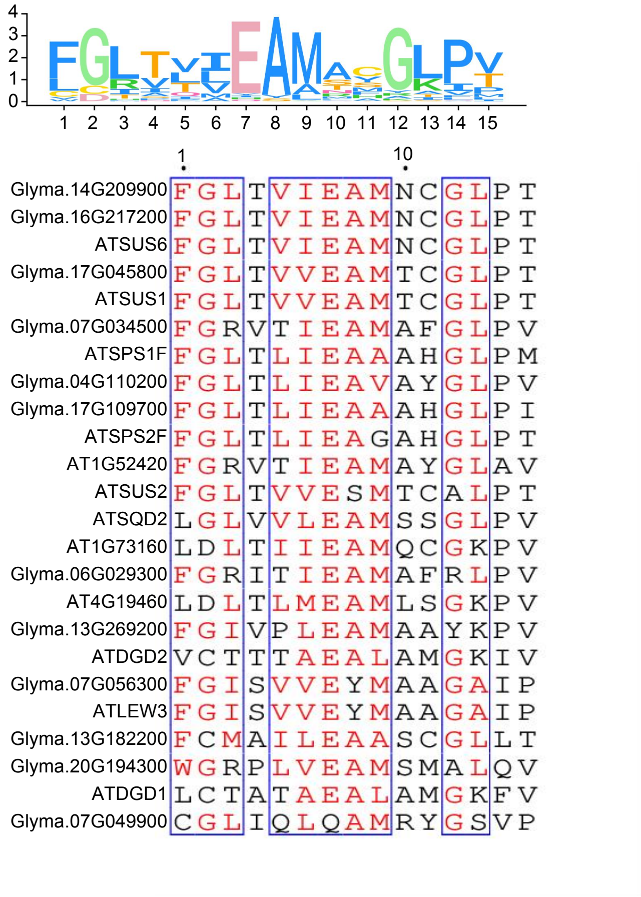


**Figure S3. Conserved motifs of genes related to the GmGT4 family.**

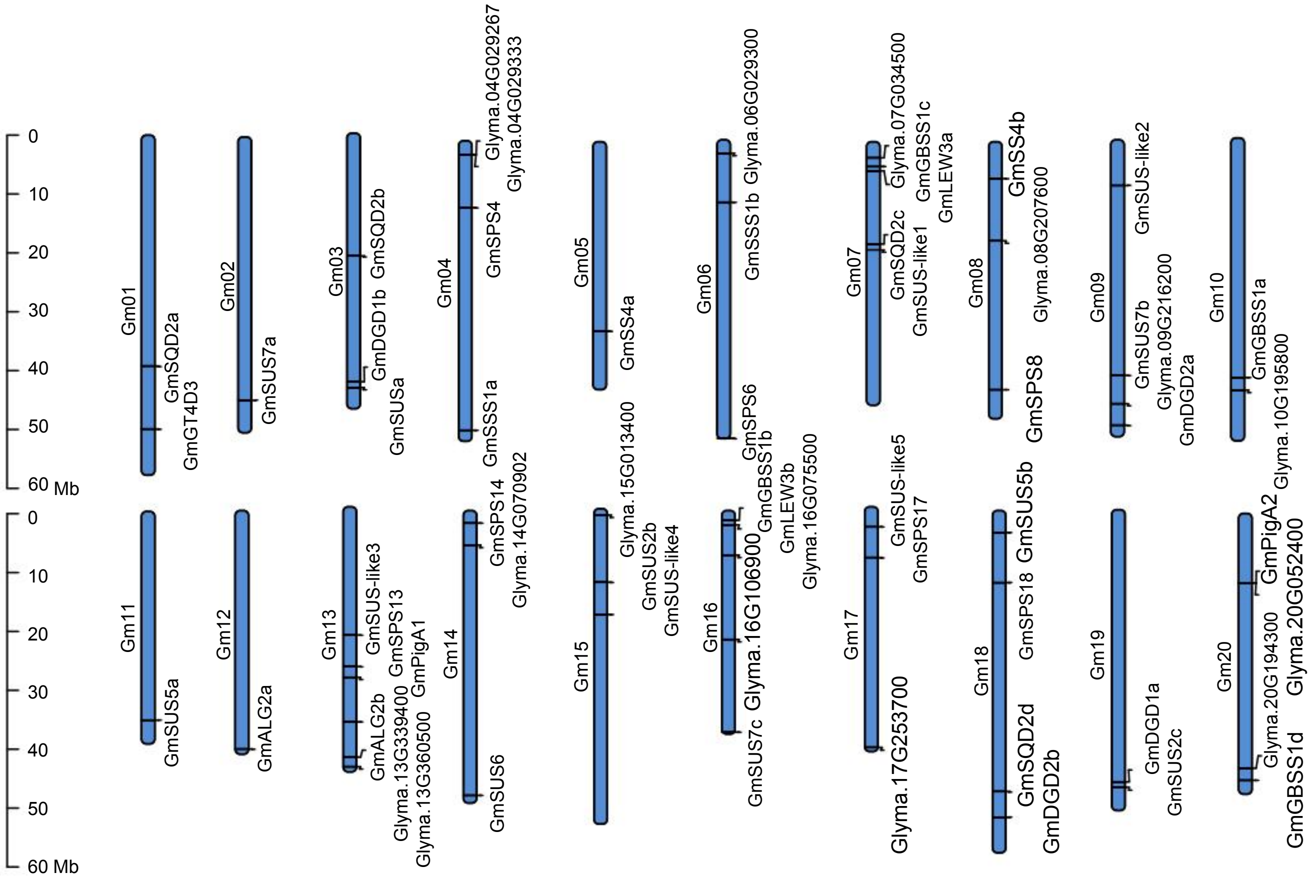


**Figure S4. Chromosomal localization of the GmGT4 family.**

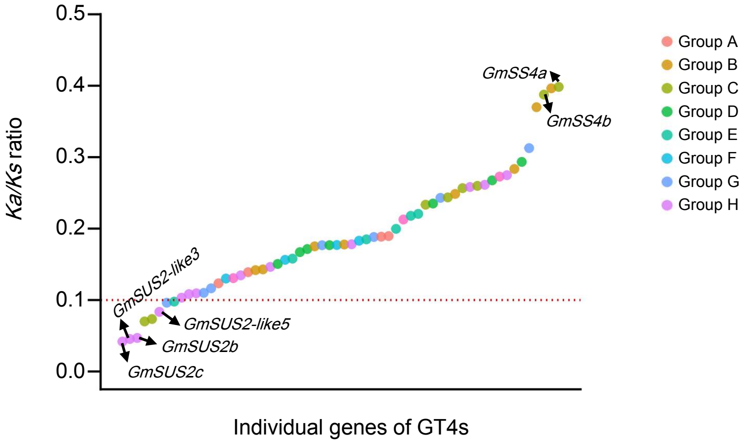


**Figure S5. Based on the selection pressure analysis of the GT4 gene family in common bean and soybean genomes.**

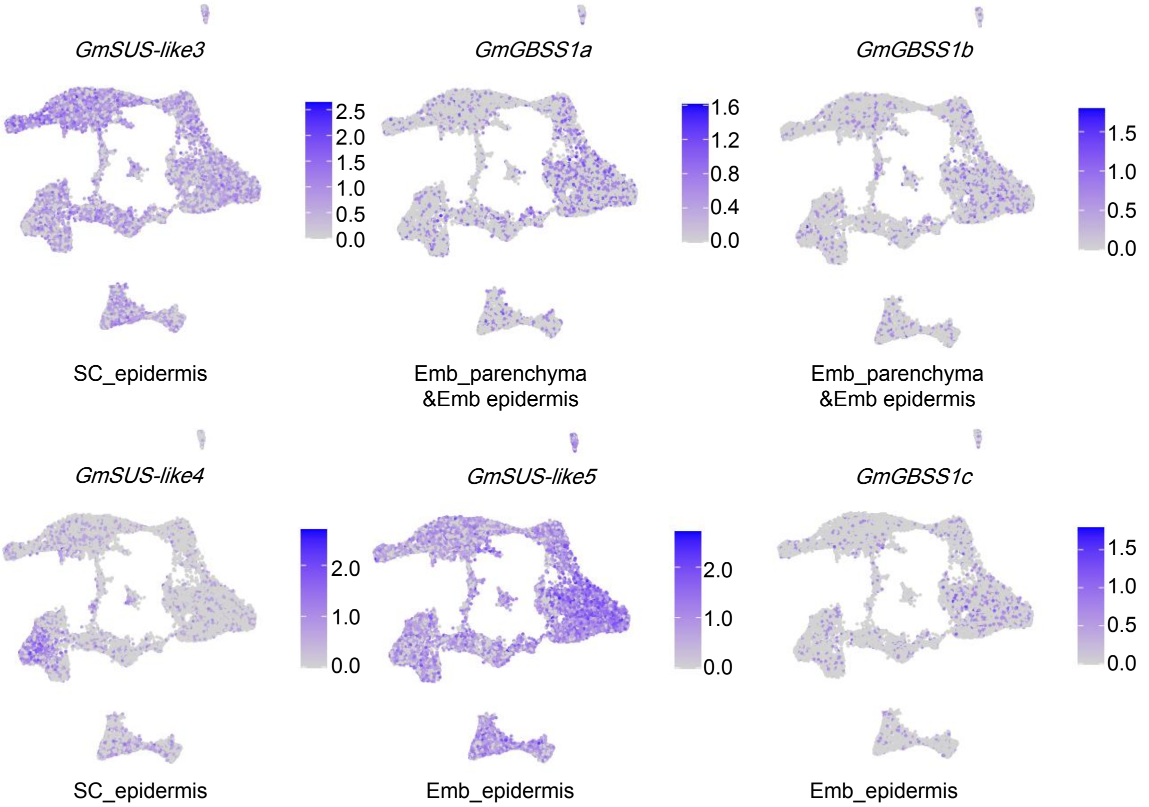


**Figure S6. Marker genes encoding GT4 family members identified from single-cell datasets of early seed maturation.**

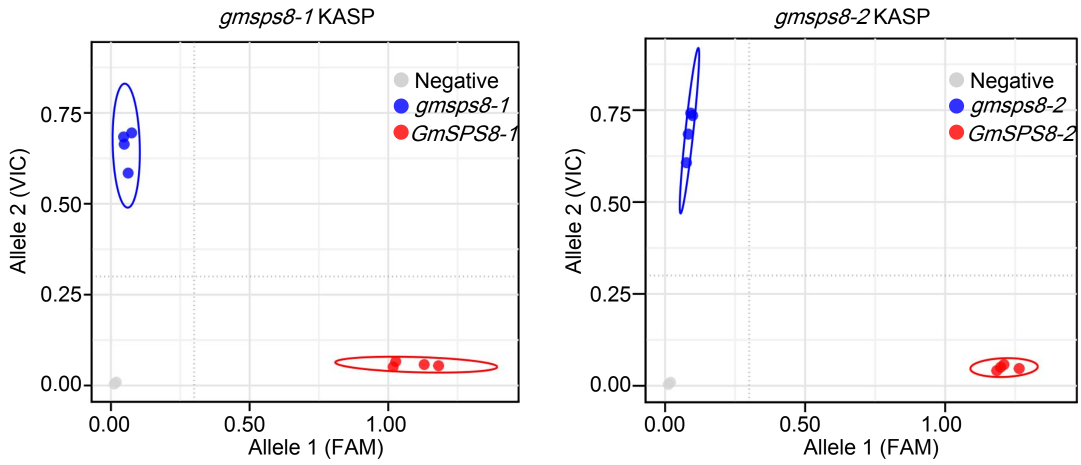


**Figure S7. Genotyping results of the GmSPS8 mutation site in EMS-mutagenized materials.**

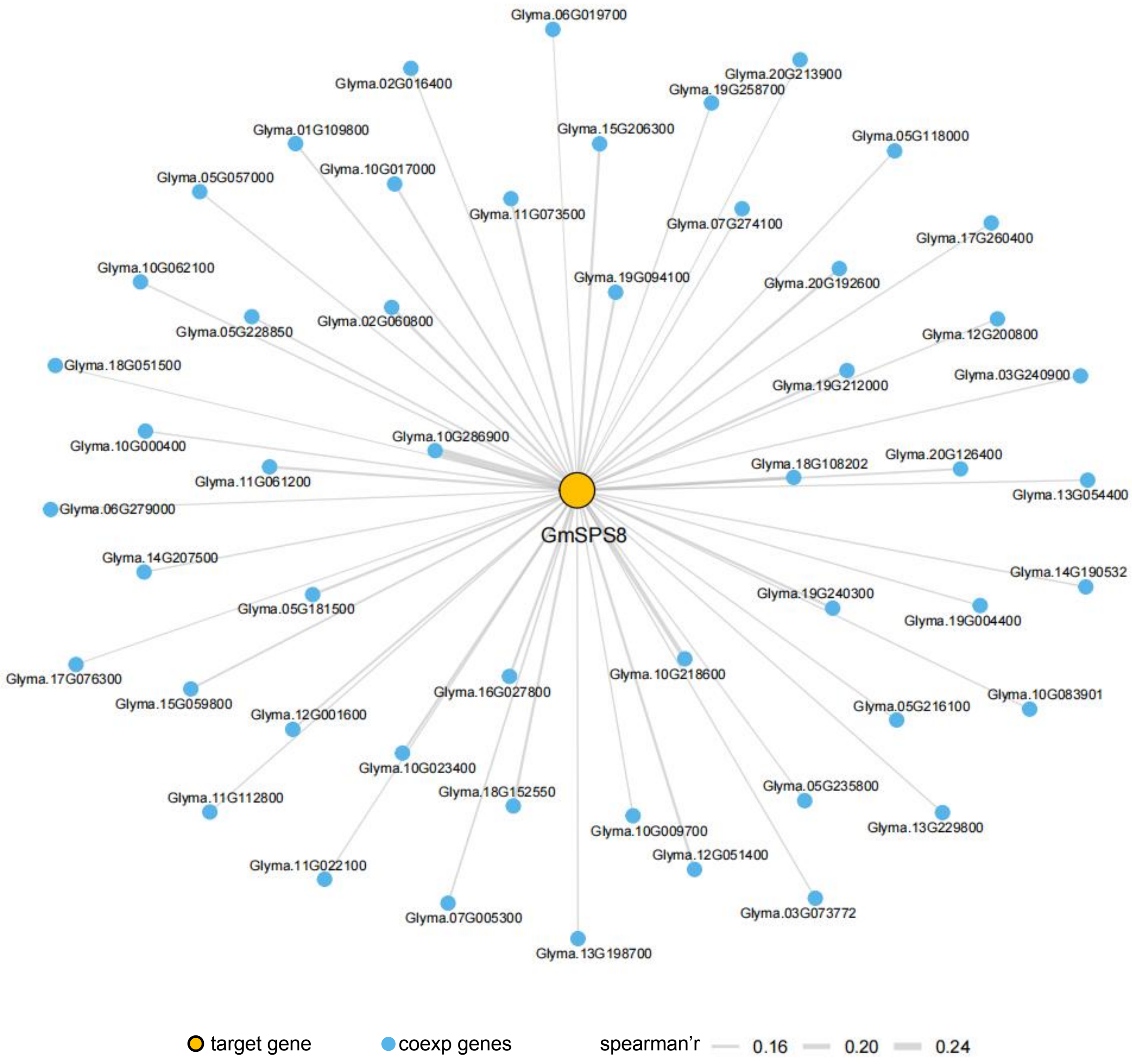


**Figure S8. Co-expression gene network of *GmSPS8* constructed based on single-cell datasets of early seed maturation.**

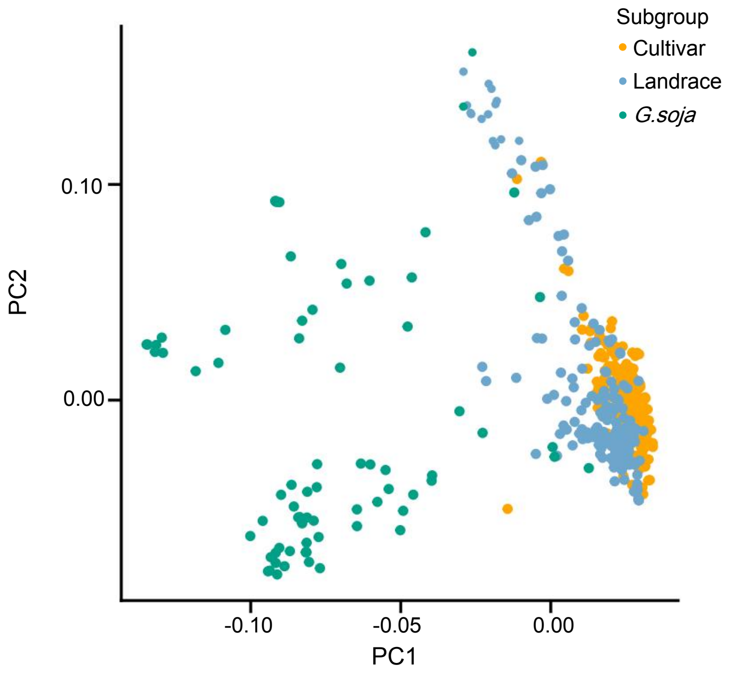


**Figure S9. Principal component analysis (PCA) of 469 accessions used for *GmSPS8* haplotype analysis.**

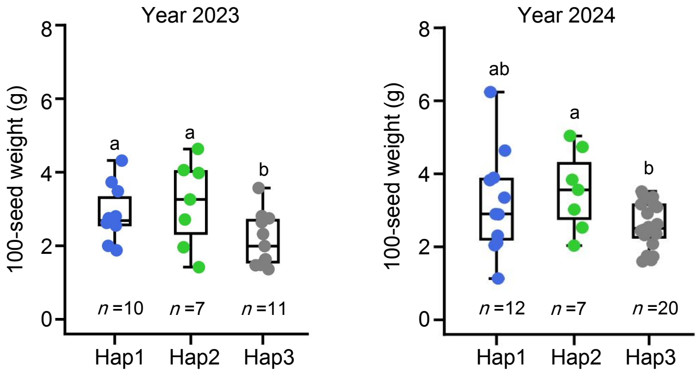


**Figure S10. Hundred-seed weight of different haplotype-specific *G.*soja germplasm in 2023 and 2024.Error bars indicate standard deviation (SD).**
